# Phytochemical composition and antioxidant properties of methanolic extracts of whole and dehulled Bambara groundnut (*Vigna subterranea*) seeds

**DOI:** 10.1038/s41598-021-93525-w

**Published:** 2021-07-08

**Authors:** Bukola C. Adedayo, Tonna A. Anyasi, Malcolm J. C. Taylor, Fanie Rautenbauch, Marilize Le Roes-Hill, Victoria A. Jideani

**Affiliations:** 1 Department of Food Science and Technology, Cape Peninsula University of Technology, Bellville, 7535 South Africa; 2 Mass Spectrometry Unit, Central Analytical Facilities, Stellenbosch University, Private Bag X1, Matieland, 7600 South Africa; 3 Applied Microbial and Health Biotechnology Institute, Cape Peninsula University of Technology, Bellville, 7535 South Africa; 4 Agro-Processing and Postharvest Technologies Division, Agricultural Research Council-Tropical and Subtropical Crops, Private Bag X11208, Nelspruit, 1200 South Africa

**Keywords:** Plant sciences, Chemistry

## Abstract

The distribution of phytochemicals and their contribution to antioxidant potentials in whole and dehulled Bambara groundnut (BGN) seeds was evaluated. Whole BGN seeds were sorted using the testa and hilium colour and further grouped into whole and dehulled BGN seeds. Extractions of both whole and dehulled BGN seeds was done using methanol and the extracts assayed for total phenolics (TPC), flavanol, flavonol, anthocyanin content, oxygen radical absorbance capacity (ORAC) and ferric reducing antioxidant power (FRAP). Methanolic extract of whole BGN seed exhibited higher flavanol and flavonol content as well as significantly higher *in-vitro* antioxidant activities than dehulled BGN seeds. The TPC of whole BGN seed extract ranged from 3.6 to 11.0 GAE/g, while that of dehulled BGN ranged from 2.7 to 3.2 GAE/g. Identification of phenolics in whole and dehulled BGN seed extract using UPLC-qTOF-MS, revealed the presence of monoterpenoids (iridoids), phenolic acids, flavonoids and lignans. Bivariate correlations showed anthocyanin demonstrated weak positive correlation between flavanol, flavonol and ORAC for whole BGN seed extract; and negative correlation between flavanol, TPC, FRAP and ORAC for dehulled BGN. Aside the effect of dehulling, whole BGN seeds exhibited the presence of phytochemicals with beneficial properties for food and industrial application.

## Introduction

Legumes are crops belonging to the *Fabaceae* family which due to the enormous protein and fibre available in their seeds, contributes a very significant and healthy portion to the human diet^1^. Apart from their nutritive worth, legumes are rich in secondary metabolites^2^ known for their contribution to a wide array of biological activities. One such legume is Bambara groundnut (BGN; *Vigna subterranea*), an underutilized neglected legume, grown for its seeds^3^. Major producers of BGN in Africa are Nigeria, Niger, Ghana and Cote d’Ivoire; but widely grown in Eastern Africa and Madagascar^4^. Likewise, in South Africa, major areas producing BGN seeds are Limpopo, Mpumalanga and KwaZulu-Natal Provinces. There are various opinions as to the origin of BGN in South Africa because the Vendas claim that they brought the legume to South Africa from Central Africa, while the people of Bolobedu also claim to have brought the seeds when they arrived in the south^5, 6^. Nevertheless, the claim by the Vendas is supported by the name ‘Nduhu-mvenda’ meaning groundnut of Venda land and the traditional ritual, which is often performed during the harvest of BGN in Venda^7^. Among the repository of underutilized crops, BGN has significant potential to ensure investment towards its improvement^8^. Its seeds possess the ability to mitigate malnutrition and increase food security as they contain about 57–67% of carbohydrate and 15–27% of proteins, although lower concentrations of lipids (< 10%) have been reported^9–12^. Bambara groundnut acts as a vital source of protein in the diets of a vast populace in Africa, especially to members of the populace that are unable to pay for animal protein. Besides, BGN is associated with diverse indigenous knowledge among cultures who depend on it for sustenance. In Limpopo Province of South Africa as well as other African countries, BGN is believed to possess medicinal value such as chewing and swallowing raw BGN to check nausea, a remedy that is used for treatment of morning illness in expectant women^4^, kidney wellness, etc. However, there is no scientific evidence to confirm these believes.

The colour of BGN seed-coats include black, red, cream/black-eye, cream/brown-eye, cream/no-eye, speckled/flecked/spotted and brown^13^. The heterogeneity of seed-coat and patterns of BGN and other legumes is credited to the occurrence of phytochemicals: secondary metabolites with biological attributes including antioxidant activity, antimicrobial effect, modulation of detoxification enzymes, stimulation of the immune system, lessening platelet aggregation and modulation of hormone metabolism and anticancer property^14, 15^. As stated by the US Food and Drug Administration (FDA), methanol is regarded as an innocuous solvent for the extraction of bioactive compounds^16^. Major groups of phytochemicals based on their chemical structure include alkaloids, sulphur-containing phytochemicals, terpenoids and polyphenols. Many researchers have reported the effect of methanolic plant extracts (1) against gastric disorders^17^; (2) on lipid profile and antioxidant status of cells^18^; (3) effect on induced diabetes in rats^19, 20^; and (4) antioxidant and phytochemical activity^21, 22^.

Nyau et al.^23^ reported that sprouting enhanced the polyphenolic profiles of red BGN. Harris et al.^24^ who studied the flavonoids and tannin composition of BGN from Mpumalanga Province of South Africa, reported that the red and brown BGN hulls contain the highest concentration of flavonoids and tannins. Flavonoid conjugates including catechin, quercetin, kaempherol, apigenin, as well as phenolic acids, saponins, sphingolipids and fatty acids, has been identified from BGN^23–25^. However, the phytochemical composition and the antioxidant activity of BGN from Limpopo Province of South Africa has not been reported. The objective of this work, was to determine the distribution of phytochemicals in BGN seeds obtained from the Limpopo Province of South Africa and their contribution to antioxidant activities.

## Materials and methods

### Chemicals

Methanol (CH_3_OH), 2,2′-azobis(2-amidinopropane) dihydrochloride (AAPH), 6-hydroxy-2,5,7,8-tetramethylchroman-2-carboxylic acid (Trolox), 2,4,6-tripyridyl-*s*-triazine (TPTZ) and aldehyde, 4-(dimethylamino)-cinnamaldehyde (DMACA). All chemicals and reagents used in this work were of analytical grade.

### Source of materials and sample preparation

The BGN seeds were purchased from an Agricultural and Food Processing Industry (NTK Foods, Makhado, Limpopo Province, South Africa) and transported to the Department of Food Science and Technology, Cape Peninsula University of Technology for sorting and further analysis.

Bambara groundnut was sorted into the different varieties of black, black-eye, brown, brown-eye and red based on the colour of the testa and hilum. A portion of sorted varieties were dehulled manually using the Corona hand mill while another group of the sorted varieties were kept whole (non-dehulled). The whole and dehulled seeds of the different varieties of black, brown, black-eye, brown-eye, red and mixture of BGN (as received) samples were milled into flour by a laboratory mill (Fritsch Pulverisette 19) with a sieve size of 0.5 mm, packed in clear ziplock plastic bags and stored in the refrigerator at 4 ℃ until further analysis. The use of BGN seeds in this study complies with international and national guidelines for the use of plant seeds in the study.

### Preparation of methanolic extracts

The methanolic extract of each variety was obtained by contacting 50 g of each flour within 500 mL of 70% (v/v) CH_3_OH overnight at 24 ℃, placed in a shaker for about 3 h, followed by centrifugation at 15,652×*g* for 15 min. The filtrate was concentrated with a vacuum evaporator until the volume was below 50 mL and then freeze-dried (35 L Genesis SQ Super XL-70, SP Scientific). The freeze-dried samples were refrigerated at 4 ℃ until further analysis.

### Oxygen radical absorbance capacity assay of Bambara groundnut methanolic extract

The oxygen radical absorbance capacity (ORAC) assay was conducted using the method of Prior et al.^26^ on a 96-well microplate using a fluorescence plate reader (Thermo Fisher Scientific, Waltham, Mass., USA). The reaction mixture comprised 12 μL of diluted BGN extract and 138 μL of fluorescein (14 μM), that was used as a target for free radical attack. The reaction was initiated by the addition of 50 μL of 768 μM 2,2′-azobis(2-amidinopropane) dihydrochloride (AAPH) and the fluorescence (emission 538 nm, excitation 485 nm) was recorded every 1 min for 2 h. Trolox was used as the standard and results were expressed as µmol Trolox equivalents (TE)/g. All analysis were carried out in triplicate.

### Ferric reducing antioxidant power of Bambara groundnut methanolic extracts

The ferric reducing antioxidant power (FRAP) assay of the BGN methanolic extracts was conducted using the method of Benzie and Strain^27^. The FRAP assay determines the ferric reducing property of antioxidants in the extracts. A total of 10 μL of the diluted BGN seed extracts was mixed with 300 μL FRAP reagent in a 96-well clear plate. The FRAP reagent consisted of a mixture of 10:1:1 (v/v/v) of acetate buffer (300 mM, pH 3.6), tripyridyl-*s*-triazine (TPTZ: 10 mM in 40 mM HCl) and FeCl_3_⋅6H_2_O (20 mM), respectively. Upon incubation at ambient temperature of 24 ℃ for 30 min, the plate was read at a wavelength of 593 nm in a Multiskan Spectrum plate reader (Thermo Fisher Scientific, USA). Ascorbic acid (AA) was used as the standard and the results were expressed as μmol ascorbic acid equivalents (AAE) per gram of sample. All tests were determined in triplicate.

### Total phenolic content of Bambara groundnut methanolic extracts

The total phenolic content (TPC) of the BGN seed extracts was determined by the method of Singleton et al.^28^. The reaction mixture comprised 25 μL of dilute BGN extracts, 125 μL of Folin Ciocalteu reagent and 100 μL of 7.5% (w/v) sodium carbonate; the mixtures were incubated for 2 h at 24 ℃ in a dark place and then measured spectrophotometrically at 765 nm using a microplate reader (Thermo Fisher Scientific, Waltham, Mass., USA) and expressed as mg gallic acid equivalents (GAE) per gram of sample. All determinations were performed in triplicate.

### Flavanol and flavonol content of Bambara groundnut methanolic extract

The flavanol content of the BGN seeds was determined colorimetrically at 640 nm using the aldehyde, 4-(dimethylamino)-cinnamaldehyde (DMACA) and expressed as mg catechin equivalents (CE) per gram of sample^29^. Flavonol content of the BGN seed extracts was determined spectrophotometrically at 360 nm using a microplate reader (Thermo Fisher Scientific, Waltham, Mass., USA) and expressed as mg quercetin equivalents (QE) per gram of sample^30^.

### Anthocyanin content of Bambara groundnut methanolic extract

The anthocyanin content of the BGN seeds was determined spectrophotometrically using the pH-differential method as stated by Wrolstad^31^. Samples of BGN extract were diluted using pH 1.0 and pH 4.5 buffers. The solutions were then allowed to equilibrate for 15 min in the dark. Absorbance for pH 1.0 and 4.5 buffers was measured at 520 and 700 nm using a microplate reader (Thermo Fisher Scientific, Waltham, Mass., USA). Monomeric anthocyanin content was calculated using the equation as stated by Wrolstad^31^. All experiments were performed in triplicate.

### Determination of metabolites present in Bambara groundnut seed extracts by ultra-performance liquid chromatography-mass spectrometry (UPLC-MS)

The UPLC-MS analysis of the BGN seed extracts was conducted using the method described by Stander et al.^32^. A Waters Synapt G2 Quadrupole time-of-flight (qTOF) mass spectrometer (MS) connected to a Waters Acquity ultra-performance liquid chromatograph (UPLC; Waters, Milford, MA, USA) was used for high-resolution UPLC-MS analysis. Column eluate first passed through a photodiode array detector (PAD) before going to the mass spectrometer, thus allowing simultaneous collection of UV and MS spectra. Electrospray ionization was used in negative mode with a cone voltage of 15 V, desolvation temperature of 275 °C, desolvation gas at 650 L/h and the rest of the mass spectrometer settings optimized for best resolution and sensitivity. Data were acquired by scanning from m/z 150 to 1500 m/z in resolution mode as well as in MSE mode. In the MSE mode, two channels of MS data were acquired: the first at a low collision energy (4 V) and the second using a collision energy ramp (40–100 V) to obtain fragmentation data as well. Leucine enkaphalin was used as reference mass for precise mass determination and the instrument was calibrated using sodium formate. Separation was done on a Waters HSS T3, 2.1 × 100 mm, 1.7 μm column. An injection volume of 2 μL was used, with the mobile phase comprising 0.1% formic acid (solvent A) and acetonitrile consisting of 0.1% formic acid as solvent B. The gradient started at 100% solvent A for 1 min and changed to 28% B over 22 min in a linear way. It then moved to 40% B over 50 s and a wash step of 1.5 min at 100% B, followed by re-equilibration to initial conditions for 4 min. The flow rate was 0.3 mL/min and the column temperature was maintained at 55 °C. Metabolites were quantified in a relative manner against a calibration curve established by injecting a range of catechin standard from 0.5 to 100 mg/L catechin. Data was processed using MSDIAL and MSFINDER (RIKEN Center for Sustainable Resource Science: Metabolome Informatics Research Team, Kanagawa, Japan).

### Statistical analysis

The results of triplicate readings were expressed as mean ± standard deviation. Statistical analysis was conducted using IBM Statistical Package for the Social Science (IBM SPSS, version 24). Obtained data were subjected to Multivariate Analysis of Variance (MANOVA) to observe the mean differences between treatments. Duncan’s multiple range test was used in separating the mean differences where differences existed. Bivariate correlation was used in establishing the relationship between BGN (forms and variety) and its antioxidant activity. The principal component analysis was used in extracting the component that explains the variability in the standardized data using cross-validation with singular value decomposition (SVD) algorithm (Unscrambler X 10.4, 2016).

## Results and discussion

### Oxygen radical absorbance capacity of Bambara groundnut extracts

**Figure 1:**
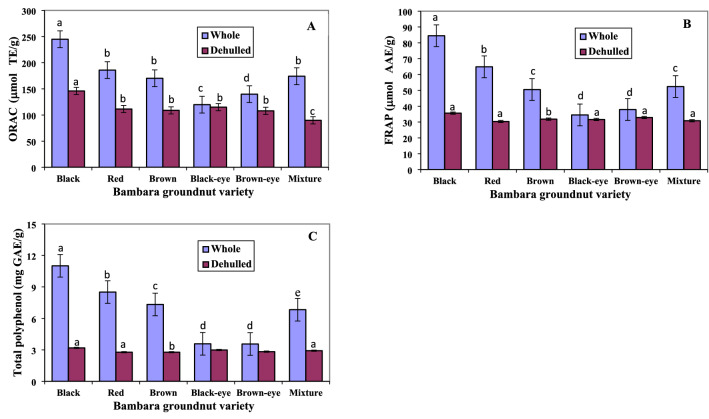
Antioxidant properties and total phenolic content of Bambara groundnut seed extracts. (**A**) Oxygen radical absorbance capacity **(**ORAC; µmol TE/g); (**B**) ferric reducing antioxidant property (FRAP; μmol AAE/g); and (**C**) total polyphenol content (mg GAE/g) of different varieties of whole and dehulled Bambara groundnut. Data represent the mean ± standard deviation of triplicate readings. Different alphabets within each form (whole or dehulled) differ significantly (p < 0.05).

The results of the oxygen radical absorbance capacity (ORAC) of both whole and dehulled BGN seed extracts expressed in micromoles of Trolox equivalents per gram of extracts are presented in Fig. 1A. There was significant difference (p < 0.05) in the antioxidant activity of the whole seed and cotyledon (dehulled) extracts except in the black-eye variety. Among the six BGN whole seed extracts, black whole seed had the highest ORAC value (244.9 μmol TE/g), followed by red (185.8 μmol TE/g), mixture (174.1 μmol TE/g), brown (170.1 μmol TE/g), brown-eye (139.9 μmol TE/g) and black-eye (119.8 μmol TE/g) whole seeds. Significant difference (p < 0.05) in ORAC was seen to exist between black and red, red and mixture, brown, brown-eye and black-eye seeds. However, no significant difference was observed between the mixture and brown as well as the brown-eye and black-eye seed extracts. Xu and Chang^33^ reported similar ORAC values for black soybean (120.4 μmol TE/g), mung bean (132.6 μmol TE/g) and adzuki bean (162.1 μmol TE/g), while Yao et al.^34^ reported higher values of 299.6 and 328.6 μmol TE/g for alkali-extractable polysaccharides from mung bean seeds. Slightly lower ORAC values were however reported by Tan and Chang^35^ on crude extracts of black bean (80.0 μmol TE/g) and black soybean (100.8 μmol TE/g) legumes. Campos-Vega et al.^36^ noted that method of processing influences the antioxidant activity in foods. However, observed changes in the antioxidant activities of processed food including legumes, could be due to the combination of several factors such as oxidative reaction, leaching, solid losses during processing as well as the development and collapse of antioxidant compositions^36^. The ORAC values for both whole and dehulled BGN seeds has not been investigated. However, extracts from the different BGN seeds used in this work showed varying antioxidant capacities in terms of the ORAC values with the whole seed extracts exhibiting higher ORAC values.

### Ferric reducing antioxidant power (FRAP) of Bambara groundnut extracts

The FRAP of the BGN extracts is shown in Fig. 1B. The black whole seed had the highest (p < 0.05) antioxidant activity (84.4 μmol AAE/g) followed by red (64.9 μmol AAE/g), mixture (52.4 μmol AAE/g), brown (50.5 μmol AAE/g), brown-eye (37.9 μmol AAE/g) and black-eye whole seed (34.5 μmol AAE/g). Oyeyinka et al.^37^ reported black and maroon BGN seed hot-water extracts showing the highest FRAP values when compared to brown and cream BGN seed hot-water extracts. Oyeyinka et al.^37^ further noted that high antioxidant activities in black and brown BGN seed extracts when compared to other seeds, could be as a result of increased concentration of protic flavonoids in the BGN seed coats. A significant difference (p < 0.05) in FRAP existed between black and red whole seed, red, mixture and brown as well as brown-eye and black-eye seed extracts. There was no significant difference in FRAP between mixture and brown whole seed and between the brown-eye and black-eye whole seed. However, significant difference (p < 0.05) was observed between the FRAP of the whole seed and cotyledon of all the varieties except for black-eye and brown-eye varieties. Yang et al.^38^ showed higher values for common edible nuts than those reported in this work, while Nyau et al.^39^ reported lower values for methanolic and aqueous extracts of brown and red BGN samples. The FRAP assay determines the capacity of antioxidants to reduce the ferric 2,4,6-tripyridyl-*s*-triazine complex [Fe (III)-(TPTZ)_2_]^2+^ to intensely blue pigmented ferrous complex [Fe(II)-(TPTZ)_2_]^2+^ in an acidic medium^39^. Ademiluyi and Oboh^40^ stated that reducing power is a potent antioxidant defense mechanism which utilizes the mechanism of electron and hydrogen atom transfer to effect this characteristics. Ferric reducing antioxidant power complex and the results are expressed as the concentration of substance having ferric-TPTZ reducing ability equivalent to that of 1 mol/L concentration of Fe (II). Mahendran et al.^41^ postulated that the reduction capacity of a compound may act as an important indicator of its potential antioxidant activity which therefore suggest that both whole and dehulled BGN posses antioxidative potentials as there was observed reduction of Fe (III) to Fe (II) in the extracts.

### Total phenolic content of Bambara groundnut

The TPC of whole and dehulled BGN seeds are shown in Fig. 1C. The black whole seed contained significantly higher (p < 0.05) total phenolics (11.0 mg GAE/g) followed by red (8.5 mg GAE/g), brown (7.3 mg GAE/g) and mixture (6.8 mg GAE/g). Brown-eye (3.6 mg GAE/g) and black-eye (3.6 mg GAE/g) had the lowest TPC. The total phenolics decreased with the lightness of the seed coat colour. Tsamo et al.^25^ showed a decrease in the TPC of BGN landraces as the colour of the seed coat decreased from darker to lighter colours. The TPC in BGN is known to be dependent on the darkness of their seed coat colour: increasing with the darkness of seed coat and decreasing with lightness. Klompong and Benjakul^42^ noted that types of extracting solvents and temperature range influences the TPC of BGN seed coat extracts. Bambara groundnut seed coats extracted with acetone at different temperatures of 30, 60 and 90 ℃ as well as ethanol at 90 ℃, were reported to possess high TPC among the different solvents and temperatures used for extraction^42^. Klompong and Benjakul^42^ and Oyeyinka et al.^37^ further postulated that recoveries of phenolics in plants are mostly reliant on the type of solvent used, solvent polarity, legume variety and method of extraction. Significant differences (p < 0.05) existed in the TPC among the black, red, brown, mixture, black-eye and brown-eye seeds. However, no significant difference was observed between the black-eye and brown-eye BGN. Significant difference (p < 0.05) was seen to exist in the total phenolics of the whole and dehulled extracts, although, no significant difference was observed among the dehulled (cotyledons) BGN varieties. The total phenolics of the whole extracts were significantly higher than dehulled BGN extracts because the outer layer of legumes is known to contain a higher concentration of phenolics due to their protective function in plants^43^. Xu and Chang^44^ in their work on legumes, stated that processing conditions leading to chemical transformation, decomposition of phenolics and the formation of phenolic-protein complex, ultimately affects the concentration of TPC in legumes.

The total phenolics obtained in this work were higher than those reported by Muchuweti et al.^45^ for BGN varieties from Zimbabwe and Ademiluyi and Oboh^40^ for fermented BGN. Similarly, the trend in the results of the total phenolics of the red, brown and other varieties reported by Muchuweti et al.^45^ compared favourably with the results obtained in this study. Marathe et al.^43^ for convenience, categorized legumes into low (below 1.0 mg GAE/g), moderate (1.0–2.0 mg GAE/g) and high (above 2.0 mg GAE/g) total polyphenol containing seeds. The concentration of total phenolics obtained in BGN extracts in this work demonstrated that the whole and dehulled BGN are high total polyphenols containing legumes. Phenolic compounds in plants are known to act effectively as antioxidants because of their chemical structure and associated redox properties. Strong correlations were observed between the TPC and the antioxidant properties of BGN seed extracts, as the black whole seed extracts exhibited the highest ORAC and FRAP values among the BGN seed extracts. Phenolic compounds are also known to exert important roles by neutralizing free radicals, chelating transitional metals and quenching singlet and triplet oxygen through delocalization or decomposition of peroxides^46^. This implies that consumption of BGN especially the whole seeds may help in combating some of the deleterious effects of free radicals.

### Flavanol, flavonol and anthocyanin content of Bambara groundnut seed extracts

The flavanol, flavonol and anthocyanin content of both whole and dehulled BGN seed extracts are shown in Table 1. The flavanol and flavonol contents in whole and dehulled BGN extracts differed significantly (p < 0.05). The whole seed was the most abundant in flavanol and the cotyledon had the least concentration. A similar trend was observed for the flavonol content. Comparatively, black whole seeds contained the highest flavanol content (p < 0.05) followed by red, brown, mixture, black-eye and brown-eye whole seeds. The flavanol content of the black whole seeds was significantly higher than other varieties (p < 0.05), whereas red whole seeds (10.2 mg QE/g) had significantly higher (p < 0.05) flavonol content than mixture (7.9 mg QE/g), black (7.7 mg QE/g), brown (6.3 mg QE/g), black-eye (5.7 mg QE/g) and brown-eye (5.7 mg QE/g) seeds.

**Table 1:** Flavanol, flavonol and anthocyanin content of dehulled and whole Bambara groundnut.

Sample	Whole	Dehulled	% reduction
**Flavanol (mg CE/g)**			
Black	2.00 ± 0.09^a^	0.28 ± 0.01^a^	86.0
Red	1.41 ± 0.01^b^	0.24 ± 0.01^ab^	83.0
Brown	1.41 ± 0.20^b^	0.22 ± 0.03^b^	84.4
Black–eye	0.36 ± 0.02^c^	0.19 ± 0.00^c^	47.2
Brown–eye	0.33 ± 0.01^c^	0.12 ± 0.02^d^	63.6
Mixture	1.04 ± 0.03^d^	0.24 ± 0.05^ab^	76.9
**Flavonol (mg QE/g)**			
Black	7.74 ± 1.20^a^	4.94 ± 0.12^a^	36.2
Red	10.24 ± 1.15^b^	5.13 ± 0.17^a^	49.9
Brown	6.33 ± 0.16^c^	4.76 ± 0.77^a^	24.8
Black-eye	5.73 ± 0.94^c^	4.99 ± 0.26^a^	12.9
Brown-eye	5.68 ± 0.40^c^	4.33 ± 0.50^b^	23.8
Mixture	7.92 ± 0.07^a^	4.62 ± 0.14^a^	41.7
**Anthocyanin content (mg/g)**			
Black	0.145 ± 0.003^a^	0.028 ± 0.005^a^	79.3
Red	0.033 ± 0.003^b^	0.030 ± 0.003^a^	9.1
Brown	0.032 ± 0.002^b^	0.030 ± 0.004^a^	6.3
Black-eye	0.032 ± 0.006^b^	0.029 ± 0.003^a^	9.4
Brown-eye	0.033 ± 0.005^b^	0.023 ± 0.004^b^	30.3
Mixture	0.038 ± 0.005^b^	0.026 ± 0.002^b^	31.6

Values represent the mean ± standard deviation of triplicate readings (n = 3).

Values with different superscript in same column are significantly different (p < 0.05).

The anthocyanin content of the whole black seed extract was the highest among the BGN seed extracts (p < 0.05). A significant difference (p < 0.05) was similarly observed for the anthocyanin concentration of the dehulled BGN extracts. Dehulling of BGN seeds resulted in 47.2–86.0% reduction in flavanol content with over 83.0% reduction in the dark coloured seeds. Reduction in flavonol was more gradual (12.9–49.9%), hence, the cotyledons retained significant amount of flavonol. However, a similar trend was not recorded for the anthocyanin content as dehulling led to between 6.3 and 79.3% reduction in anthocyanin concentration. The black seed BGN extract was most affected by dehulling with 79.3% reduction of anthocyanin content.

Anthocyanins, flavanols and flavonols are members of the subclass of flavonoids, a plant secondary metabolite generally synthesized by the shikimate pathway, where they are formed from intermediates of carbohydrate metabolism^47^. Flavonoids are the largest class of dietary polyphenols that acts as reducing agents, metal chelators, reactive oxygen species (ROS) scavengers, chain-breaking antioxidants and quenchers of singlet oxygen formation. As signaling molecules, flavonoids interact with crucial cellular receptors or proteins (kinases and enzymes) cascades to catalyze or control signaling or regulatory pathways, leading to physiological responses or gene expression^48^. Consumption of foods rich in flavonoids has therefore shown to help prevent some chronic diseases and provide therapeutic effects.

### Ultra performance liquid chromatography-mass spectrometry (UPLC-MS) determination of metabolites in Bambara groundnut seed extracts

The phenolic compounds identified from both whole and dehulled BGN methanolic extracts are shown in Table 2. Flavonoids, phenolic acids, lignans and monoterpenes were identified in the BGN extracts based on mass spectra obtained in the negative mode using their fragmentation pattern and data from published literature. However, the most represented classes of polyphenolic compounds in the BGN extracts were flavonoids with a total of five compounds. Most of the phenolic compounds in BGN is more abundant in the testa than in the cotyledon.

**Table 2:** Metabolites identified by LC–MS in negative ionisation mode present in Bambara groundnut seed methanolic extracts.

Peak	Rt (min)	λ_max_ (nm)	[M–H]^−^	Formula	Error (mDA)	[M–H]^−^ fragments	Tentative identification	Classification
1	7.66	260	495.137	C_19_H_27_O_15_	3	485, 323, 125, 101	Unknown	
2	8.28	Weak	305.088	C_12_H_17_O_9_	2.3	259	Starch acetate	Oligosaccaride
3	8.37	Weak	421.135	C_17_H_25_O_12_	0	259, 161	Lamiide	Monoterpenes (Iridoid O-glycoside)
4	9.20	278	451.126	C_21_H_24_O_11_	1.5	289, 245, 134	Catechin hexoside	Flavonoid
5	9.74	Weak	205.071	C_8_H_13_O_6_	4.2	187, 151	Diethyl tartrate	beta hydroxy acids and derivatives
6	10.67	280	577.135	C_30_H_25_O_12_	0.9	407, 289, 161	Procyanidin dimer B1	Flavonoid
7	11.07	Weak	485.187	C_19_H_34_O_14_	− 2.5	409, 455	Caffeic acid derivative	Phenolic acid
8	11.32	270	451.124	C_21_H_23_O_11_	− 0.2	289, 245	Catechin hexoside	Flavonoid
9	11.92	Weak	323.134	C_13_H_23_O_9_	− 1.9	252, 161	Beta-d-glucosyl-(1->3)-alpha-l-fucosyl	O-Glycosyl compounds
10	12.06	Weak	691.177	C_35_H_31_O_15_	− 3.6	572, 447, 323, 281, 259	UNPD69159	Neolignans and related compounds
11	13.69	Weak	387.164	C_18_H_27_O_9_	− 3.4	207	Medioresinol	Lignan
12	15.87	353	609.147	C_27_H_29_O_16_	2.1	300, 271	Quercetin 3-galactoside 7-rhamnoside	Flavonoid
13	16.32	Weak	381.191	C_20_H_29_O_7_	1.3	337, 291	Caffeic acid derivative	Phenolic acid
14	17.82	350	463.089	C_21_H_19_O_12_	2.2	300, 271, 255	Quercetin hexoside	Flavonoid
15	23.89	250	655.299	C_36_H_46_NO_11_	− 0.26	551, 313, 143	UNPD205773	O-Glycosyl compounds

*Rt* retention time.

**Figure 2:**
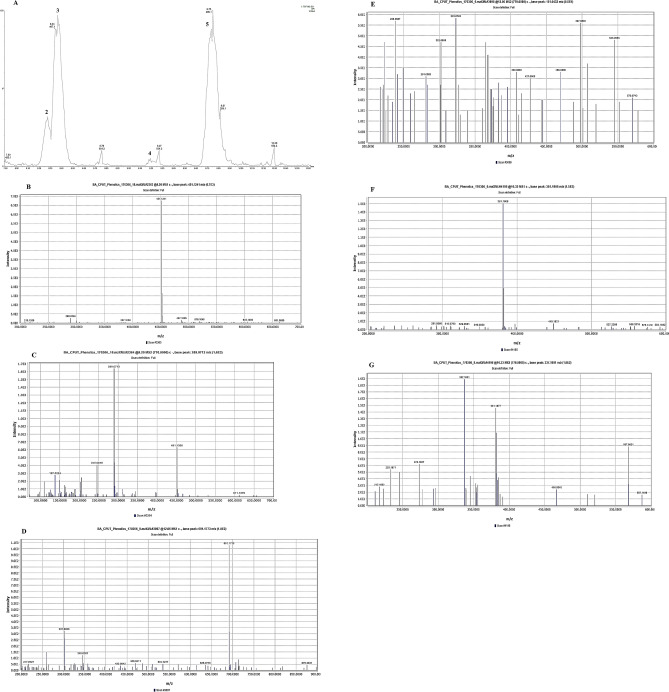
Phytochemicals and mass spectra of identified flavonoids and phenolic acids in Bambara groundnut samples. (**A**) chromatogram of identified compounds; (**B**) unfragmented spectra MS1 of compound **4** (m/z 451 and Rt, 9.20); (**C**) fragmentation spectra MS2 of compound **4** in negative (ESI−) mode; (**D**) unfragmented MS1 spectra of compound **10** (m/z 691 and Rt, 12.06); (**E**) fragmentation spectra MS2 of compound **10** in negative (ESI−) mode; (**F**) unfragmented MS1 spectra of compound **13** (m/z 381 and Rt, 16.32); (**G**) fragmentation spectra MS2 of compound **13** in negative (ESI−) mode.

Compound **1** with retention time (Rt) of 7.66 was unidentified, while compound **3** having a molecular weight of 421.135 was classified as a monoterpene. Compounds **4** and **8** having molecular weights of 451.126 and 451.124 and Rt of 9.20 and 11.32 were identified as catechin hexoside. Both compounds fragmented to m/z 289 (Fig. 2A–C) and 245, with compound **4** further fragmenting to m/z 134. Similarly, compound **6** was identified as procyanidin dimer B1; compound **12** as quercetin 3-galactoside 7-rhamnoside; and compound **14** as quercetin hexoside are flavonoids present in the BGN extracts. Compounds **7** and **13** were identified as caffeic acid derivatives with Rt of 11.07 and 16.32 (Fig. 2D–G) while compound **5** is a quinic acid derivative. Adebiyi et al.^49^ reported the presence of compounds **3, 7, 11** and **13** in *Dawadawa,* a fermented BGN condiment. Compound **3,** tentatively identified as lamiide is an iridoid glycoside. Iridoids are monoterpenoids present in plants and animals and which due to their unstable nature, mostly combine with sugars to form glycosides in plants^50, 51^. Iridoids are stated to confer biological properties in the body such as liver protection, anti-inflammatory, anti-tumor and cholagogic effects, hypoglycemic, hypolipidemic, cardiocerebrovascular activity, and alpha-glucosidase activity^51–53^. Tsamo et al.^25^, identified compounds **4, 6** and **8** from 21 BGN landraces obtained from South Africa, Swaziland and Mozambique. Nyau et al.^54^ further reported the presence of compound **14** in BGN samples cooked domestically for 410–430 min. Compound **14** with Rt of 17.82, and tentatively identified as quercetin 3-galactoside is a monoglycoside of quercetin previously identified in cowpea phenotypes^55^.

Awika and Duodu^50^ described the extractable phenolic acids occurring in cowpea and pulses as esters of *p-*hydrobenzoic acid, caffeic acid, gallic acid and syringic acid. Variations in the concentration of these phenolic acids was reported to be dependent on the legume phenotype. However, cowpeas having red seed coats were seen to possess a higher phenolic acid concentration^56^. Ojwang et al.^55^ postulated that flavonoids are largely concentrated in the seed coat of pulses, with their effect on the colour of the seed coat of great influence on their choice and use in different food applications. Consumption of pulses due to the availability of bioactive compounds have been implicated for the reduction of cardiovascular disease risk factors^57^. Furthermore, a diet rich in pulses provides a significant amount of phenolic acids, carotenoids and tocopherols that acts as a shield for low-density lipoprotein cholesterol against free radical oxidation^57^.

### Correlations between antioxidant activity and phytochemicals in whole and dehulled Bambara groundnut

Bivariate correlation (Table 3) showed that in whole BGN, a significantly strong positive relationship exists between the flavanol, total polyphenol (r = 0.967, p = 0.000), FRAP (r = 0.896, p = 0.000) and ORAC (r = 0.668, p = 0.000) with a moderate relationship with flavonol and anthocyanin. Flavonol had strong positive correlation with FRAP (r = 0.594, p = 0.0010; total polyphenol (r = 0.559, 0.002) and a moderate relationship with ORAC (r = 0.469, r = 0.014). Anthocyanin had a significantly strong positive relationship with total polyphenol, FRAP and ORAC. In dehulled BGN seeds, a weak relationship existed between the flavanol and the total polyphenol content (r = 0.0941, p = 0.000); a significantly (p < 0.05) strong positive relationship between ORAC (r = 0.641, p = 0.000) and FRAP (r = 0.571, p = 002); and a significantly negative relationship between flavanol and anthocyanin content (r = − 0.594, r = 0.001). A significantly strong relationship existed between anthocyanin, total polyphenol and FRAP. However, for both the whole and dehulled seeds, anthocyanin concentration showed weak positive correlation between flavanol, flavonol and ORAC antioxidant activity for the whole BGN seed methanolic extract, while negative correlation was observed between anthocyanin concentration and flavanol, TPC, FRAP and ORAC antioxidant activity. Negative correlations observed between anthocyanin and other phytochemicals in the dehulled BGN seeds, could be attributed to the effect of dehulling. The correlation of the phytochemicals with the TPC and the antioxidant activities in both the whole and dehulled seeds could imply that the relatively high antioxidant activity of the methanolic extract of BGN might be as a result of its high phenolics which act as free radical scavengers or primary antioxidant^25^. Thus, methanolic extracts of BGN may prove to be beneficial (1) against gastric disorders^17^; (2) on the lipid profile and antioxidant status of cells^15^; (3) on induced diabetes in rats^19, 20^; and (4) on antioxidant and phytochemical activity^21, 22^.

**Table 3:** Bivariate correlation between the phytochemicals and antioxidant activity of Bambara groundnut.

	Flavanol	Flavonol	Anthocyanin
**Undehulled**			
Flavonol	0.471 (0.013)*		
Anthocyanin	0.385 (0.047)*	0.109 (0.587)	
Total polyphenol	0.967 (0.000)**	0.559 (0.002)**	0.520 (0.005)**
FRAP	0.896 (0.000)**	0.594 (0.001)**	0.629 (0.000)**
ORAC	0.668 (0.000)**	0.469 (0.014)*	0.0782 (0.000)
**Dehulled**			
Flavonol	0.265 (0.181)		
Anthocyanin	− 0.594 (0.001)**	0.034 (0.867)	
Total polyphenol	0.941 (0.000)**	0.352 (0.072)	− 0.514 (0.006)**
FRAP	0.571 (0.002)**	− 0.051 (0.801)	− 0.423 (0.028)*
ORAC	0.641 (0.000)**	0.382 (0.049)*	− 0.258 (0.195)

Correlation coefficient and p-value in bracket. Correlation is significant at the 0.05 level (*) and 0.01 level (**) (two-tailed).

**Figure 3:**
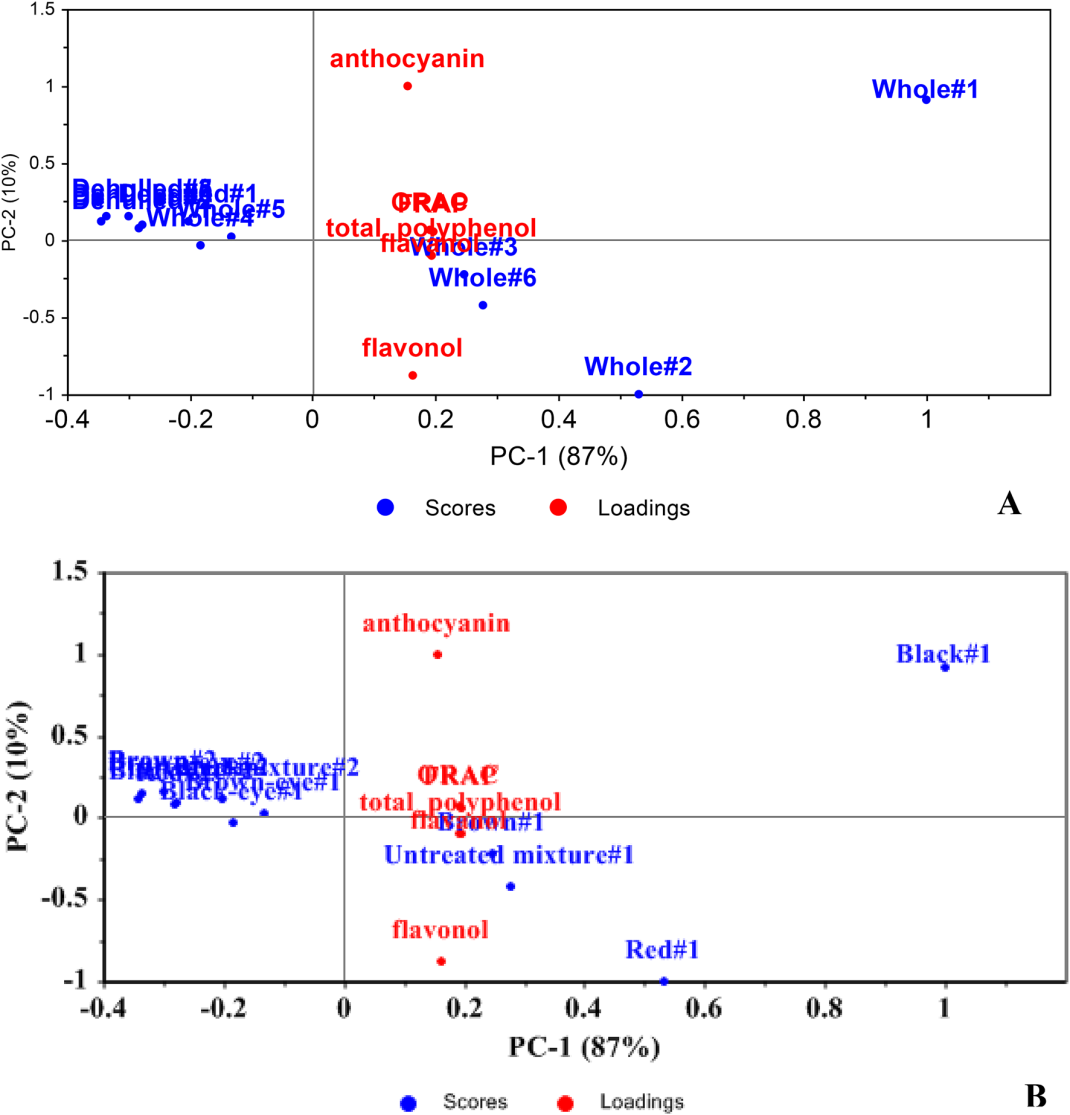
Principal component scores and loadings based on Bambara groundnut (**A**) form, (**B**) variety with respect to phytochemicals and antioxidant activity.

The principal components on BGN form and varieties concerning their phytochemicals and antioxidant activity is detailed in Fig. 3. The variation in the data can be explained by two principal components (PC), with PC1 explaining 87% and PC2 10% of the variation (Fig. 3A,B). Dehulling reduced the phytochemicals and consequently the antioxidant activity of BGN. The whole black variety was correlated with high anthocyanin and the whole red, brown and mixed varieties were high in flavonol. It appears that the FRAP, ORAC, flavanol and total polyphenol could not be used in categorising the BGN varieties. Although dehulling reduced the phytochemicals in BGN, its concentration in the cotyledon was sufficient as there was no observed discrimination between the dehulled and the whole. This is of interest as the consumption of dehulled BGN could still provide some phytonutrients to the consumer.

## Conclusions

The distribution of phytochemicals and antioxidant activities in the BGN methanolic extracts varied with the testa and hilium colour. Phenolic acids, flavonoids and lignan were identified in the whole and dehulled BGN seeds at varying concentrations. The whole seeds of BGN exhibited a higher concentration of phytochemicals (total polyphenolics, flavanol, flavonol and anthocyanins) when compared to their cotyledons, with the BGN seed hulls exhibiting higher antioxidant activities. Higher polyphenolic content and thus antioxidant activities in whole seeds can be attributed mostly to the outer layer of legumes containing greater concentrations of phenolics as a result of their protective function in plants. However, dehulling was observed to reduce the concentration of phytochemicals in BGN seeds, although minimal effects was seen in the concentration of phytochemicals in the cotyledon. The presence of phenolics which was higher in the dark coloured and whole seeds, demonstrated the potential of BGN among other legumes and pulses, with tremendous beneficial properties for food and industrial applications.
